# C-Reactive Protein–Albumin–Lymphocyte Index and the Modified Glasgow Prognostic Score as Predictors of Early Mortality After Palliative Percutaneous Transhepatic Biliary Drainage in Malignant Biliary Obstruction

**DOI:** 10.3390/jcm15124608

**Published:** 2026-06-13

**Authors:** Hatice Ayyıldız Sevim, Kadriye Bir Yücel, Galip Can Uyar, Hayriye Şahinli

**Affiliations:** Department of Medical Oncology, Ankara Etlik City Hospital, Ankara 06170, Türkiye; kadriyebiryucel@gmail.com (K.B.Y.); g.can_uyar@hotmail.com (G.C.U.); dr.hayriye@hotmail.com (H.Ş.)

**Keywords:** percutaneous transhepatic biliary drainage, malignant biliary obstruction, early mortality, CALLY index, modified Glasgow Prognostic Score

## Abstract

**Background**: Biliary drainage is a key component of palliative management in patients with malignant biliary obstruction. In cases where endoscopic approaches are unsuccessful or cannot be performed, percutaneous transhepatic biliary drainage (PTBD) represents an established alternative for achieving biliary decompression. The C-reactive protein–albumin–lymphocyte (CALLY) index combines inflammatory, nutritional, and immune-related parameters into a single marker, while the modified Glasgow Prognostic Score (mGPS), based on C-reactive protein and albumin concentrations, reflects the systemic inflammatory status of the patient. This study aimed to evaluate the prognostic value of the preprocedural CALLY index and mGPS in predicting 30-day mortality among patients with advanced malignant biliary obstruction undergoing palliative PTBD. **Methods**: This single-center retrospective study was conducted in a total of 179 patients who underwent palliative PTBD for malignant biliary obstruction at Ankara Etlik City Hospital between December 2022 and June 2025. **Results**: The 30-day mortality rate was 25.1%. The cut-off value for CALLY was determined as 67 based on receiver operating characteristic (ROC) curve analysis, and mGPS was categorized as 0–1 versus 2. In univariable Cox regression analyses, pancreaticobiliary tumor type, mGPS = 2, and CALLY < 67 were associated with early mortality. In multivariable Cox analysis, CALLY ≥ 67 was independently associated with a reduced risk of 30-day mortality, whereas pancreaticobiliary tumor type was independently associated with an increased risk. In the CALLY–mGPS risk stratification, 30-day mortality rates were 8.0%, 13.5%, and 44.1% in the low-, intermediate-, and high-risk groups, respectively. **Conclusions**: In this retrospective cohort, preprocedural inflammation- and nutrition-based markers were found to be associated with early mortality in patients with malignant biliary obstruction undergoing PTBD. Accordingly, risk stratification using readily available parameters such as CALLY and mGPS appears feasible in the preprocedural setting. The CALLY–mGPS-based approach may provide a practical framework for clinical risk assessment; however, prospective multicenter validation, including tumor-specific subgroup analyses, is warranted.

## 1. Introduction

Malignant biliary obstruction develops when tumor involvement of the biliary tract or extrinsic compression by adjacent malignant disease disrupts normal bile flow [1]. The etiological spectrum of malignant biliary obstruction is broad and includes both primary pancreatobiliary malignancies and secondary involvement from metastatic disease. Common causes include pancreatic adenocarcinoma, cholangiocarcinoma, gallbladder cancer, hepatic metastases, ampullary or duodenal tumors, gastric cancer, hepatocellular carcinoma, and metastatic lymphadenopathy leading to hilar compression [2,3]. In the vast majority of patients, malignant obstructive jaundice is not amenable to curative treatment and is associated with a poor prognosis [4].

Percutaneous transhepatic biliary drainage (PTBD) is a commonly used decompression method in malignant biliary obstruction [5]. Biliary drainage can be achieved by surgical drainage, endoscopic retrograde cholangiopancreatography (ERCP), or PTBD; however, no definitive superiority has been demonstrated among these approaches [6]. In clinical practice, PTBD is frequently preferred for proximal obstructions caused by malignant lesions above the level of the common hepatic duct, whereas distal biliary obstructions are generally managed with ERCP or endoscopic ultrasound-guided biliary drainage [7].

PTBD is considered a safe and effective procedure; however, it is not without risk [8]. Complications, particularly sepsis, are common, with reported 30-day mortality and procedure-related complication rates of 19.8% and 61.7%, respectively [9]. In some patient cohorts, overall survival after PTBD has been reported to be as short as 48 days [10]. Given the clinical heterogeneity of the patient population, early identification of patients with poor prognosis is crucial to avoid unnecessary procedural risks, prolonged hospitalization, and early mortality.

Systemic inflammation and nutritional status are increasingly recognized as key determinants of cancer prognosis. The prognostic significance of the modified Glasgow Prognostic Score (mGPS), which integrates C-reactive protein (CRP) and albumin, has been validated across a range of malignancies, including pancreatic cancer, biliary tract cancer, and colorectal cancer [11,12,13]. Similarly, an increased CRP/albumin ratio (CAR) has been consistently associated with poorer overall survival in patients with cancer [14]. Moreover, the Prognostic Nutritional Index (PNI), which is based on serum albumin levels and lymphocyte count, has been shown to be associated with clinical outcomes—particularly in gastrointestinal and pancreatic cancers—and lower PNI values have been linked to an unfavorable prognosis [15,16]. The Systemic Immune-Inflammation Index (SII), which incorporates neutrophil, lymphocyte, and platelet counts, has also been investigated as an indicator of the balance between systemic inflammation and immune response, with prognostic relevance reported in several solid malignancies [17]. The C-reactive protein–albumin–lymphocyte (CALLY) index is a composite marker that captures systemic inflammation, nutritional reserve, and lymphocyte-mediated immune status. Recent studies in gastrointestinal malignancies have suggested that reduced CALLY values are associated with less favorable survival outcomes in gastric, colorectal, esophageal, and pancreatic cancers [18]. Taken together, these markers provide a measurable overview of the patient’s systemic inflammatory and nutritional condition and may help estimate prognosis and clinical outcomes.

To date, the prognostic value of inflammation- and nutrition-based indices in patients undergoing PTBD for malignant biliary obstruction has been evaluated to a limited extent. To substantiate this research gap, we conducted a focused literature search in PubMed, Web of Science, and Scopus using combinations of the terms “CALLY index”, “C-reactive protein albumin lymphocyte index”, “percutaneous transhepatic biliary drainage”, “PTBD”, “malignant biliary obstruction”, and “30-day mortality”. No previous study was identified that specifically evaluated the association between the CALLY index and 30-day mortality in patients with malignant biliary obstruction undergoing PTBD. In this patient population, clarifying the relationship between inflammatory and nutritional status and clinical characteristics and survival is important for early risk stratification. This study primarily aimed to determine whether preprocedural CALLY and mGPS could predict 30-day mortality in patients undergoing PTBD for malignant biliary obstruction. In addition, we aimed to investigate the ability of a CALLY–mGPS-based risk classification to meaningfully discriminate patients according to early mortality risk.

## 2. Materials and Methods

### 2.1. Study Design and Patient Selection

In this retrospective single-center cohort, we evaluated patients who underwent palliative PTBD for malignant biliary obstruction at Ankara Etlik City Hospital between December 2022 and June 2025. Patients were eligible if they had histopathologically confirmed malignancy, received PTBD for biliary obstruction, and were not planned for curative resection because of advanced disease, an unsuitable clinical condition for surgery, or refusal of surgical treatment. Technical success was considered achieved when the targeted biliary system was accessed percutaneously and a drainage catheter and/or stent was placed appropriately, allowing biliary drainage during the procedure. Patients with non-malignant obstructive etiologies, those who underwent surgical treatment for biliary obstruction after PTBD, cases without definitive histologic confirmation of malignancy, and patients with unavailable survival data were excluded.

Thirty-day mortality was evaluated as death from any cause within 30 days after the PTBD procedure. Mortality status was ascertained by reviewing electronic patient records and institutional follow-up data.

### 2.2. Data Collection

Study data were obtained through a retrospective review of the hospital-based digital patient record system and imaging archives. For each patient, demographic and clinical variables were collected, including age, sex, primary tumor type, previous ERCP or surgical history, metastatic status, and systemic treatment information. Laboratory assessment comprised complete blood count parameters obtained pre-procedurally and on post-procedure day 7, together with routine biochemistry tests representing markers of inflammation, nutritional status, and cholestasis. Using these data, inflammation- and nutrition-based indices were calculated according to definitions in the literature: PNI = serum albumin (g/L) + 5 × absolute peripheral lymphocyte count (×10^9^/L); CAR = CRP (mg/L)/albumin (g/L); SII = platelet count × neutrophil count/lymphocyte count. The CALLY index was calculated as (serum albumin [g/L] × absolute lymphocyte count [×10^9^/L])/CRP [mg/L]. The modified Glasgow Prognostic Score (mGPS) was assigned as 0–2 based on CRP and albumin levels: mGPS 0 (CRP ≤ 10 mg/L and albumin ≥ 35 g/L), mGPS 1 (CRP > 10 mg/L or albumin < 35 g/L), and mGPS 2 (CRP > 10 mg/L and albumin < 35 g/L).

### 2.3. Ethical Approval

The study protocol was approved by the Clinical Research Ethics Committee of Ankara Etlik City Hospital (Date: 14 May 2025; Approval No: AEŞH-BADEK-2025-129). The study was conducted in accordance with the principles of the Declaration of Helsinki.

### 2.4. Procedural Details

Percutaneous transhepatic biliary drainage was performed by experienced interventional radiologists as part of the multidisciplinary management of patients with malignant biliary obstruction. The choice of biliary decompression technique, including external or internal drainage, metal stent placement, or a combination of these approaches, was determined according to the anatomical complexity of the obstruction and the overall clinical treatment strategy.

In some patients, repeated drainage procedures were required due to inadequate biliary decompression or disease progression. Data related to all procedures were retrieved from the hospital electronic medical record system and analyzed.

### 2.5. Statistical Analysis

Continuous variables were summarized as mean ± standard deviation (SD) or median with range, as appropriate, while categorical variables were presented as counts and percentages. Normality of continuous variables was assessed using visual inspection and distributional methods. Pre- and post-PTBD laboratory values were compared with either the paired-samples *t*-test or the Wilcoxon signed-rank test, according to the distributional characteristics of the data.

The primary endpoint was 30-day mortality, defined as death from any cause within 30 days after the PTBD procedure. Associations between baseline variables and 30-day mortality were initially explored using univariable analyses. Categorical variables were compared using the chi-square test or Fisher’s exact test, as appropriate.

Time-to-event analyses for 30-day mortality were performed using Cox proportional hazards regression models. Variables with clinical relevance and/or a *p* value < 0.10 in univariable analyses were considered for inclusion in multivariable models. Because several inflammation- and nutrition-based indices shared common laboratory components, particularly CRP and/or albumin in CAR, mGPS, and the CALLY index, entering these variables together in the same model could introduce statistical dependency. Therefore, parameters with substantial component overlap were not included concurrently in multivariable analyses. Potential multicollinearity was examined using the variance inflation factor (VIF), and model structures with VIF values greater than 5 were considered unstable and were not retained in the final analysis. A parsimonious clinically adjusted multivariable Cox model was constructed.

The discriminative ability of CAR, SII, and the CALLY index for 30-day mortality was evaluated using receiver operating characteristic (ROC) curve analysis. For each index, the area under the curve (AUC) and corresponding 95% confidence intervals (CIs) were calculated using the DeLong method. For each marker, the cut-off value was chosen using the Youden index, which maximizes the combined performance of sensitivity and specificity. To examine the internal validity of the AUC estimates, bootstrap resampling with 1000 iterations was performed. Sensitivity, specificity, positive predictive value, negative predictive value, and accuracy were calculated at the selected thresholds. The reporting of the CALLY–mGPS risk stratification model was guided, where applicable, by the Transparent Reporting of a Multivariable Prediction Model for Individual Prognosis or Diagnosis (TRIPOD) statement [19].

All statistical tests were two-sided, and a *p* value < 0.05 was considered statistically significant. Statistical analyses were performed using IBM SPSS Statistics for Windows, version 26.0 (IBM Corp., Armonk, NY, USA).

## 3. Results

### 3.1. Patient Characteristics

After applying the eligibility criteria, 179 patients treated with PTBD for malignant biliary obstruction were analyzed. Male patients constituted 53.6% of the cohort. At the time of PTBD, pancreatic cancer (36.3%) and cholangiocarcinoma/gallbladder cancer (26.3%) were the most frequent primary tumor types. At the time of PTBD, all patients had stage IV disease. When metastatic disease patterns were evaluated, liver-only metastasis was the most common presentation (57.0%). The median overall survival for the entire cohort was 6.74 months (95% CI, 4.63–8.84).

Within 30 days after PTBD, 45 patients (25.1%) died. Baseline characteristics according to 30-day mortality status are presented in Table 1. The table includes demographic and clinical variables, primary tumor types, laboratory parameters, inflammation- and nutrition-based indices, and procedure- and treatment-related factors.

PNI and mGPS were classified using previously established thresholds from the literature. For CALLY, CAR, and SII, thresholds were identified by ROC curve analysis. The ROC-based thresholds and AUC findings are shown in Table 2.

### 3.2. Changes in Laboratory Parameters After PTBD

Paired analyses demonstrated significant improvements in cholestatic parameters following PTBD. Both total and direct bilirubin levels decreased markedly after the procedure (both *p* < 0.001). Liver enzymes including alanine aminotransferase (ALT), aspartate aminotransferase (AST), gamma-glutamyl transferase (GGT), and alkaline phosphatase (ALP) also showed significant reductions (all *p* < 0.001). In contrast, inflammatory markers increased after PTBD, with a significant rise in white blood cell count (*p* < 0.001), neutrophil count (*p* = 0.014), and C-reactive protein (CRP) levels (*p* < 0.001). Changes in lymphocyte counts did not reach statistical significance (*p* = 0.063). Post-procedural laboratory parameters were recorded on day 7 after PTBD. Pre- and post-PTBD laboratory values are presented in Table 3.

### 3.3. Univariable and Multivariable Cox Regression Analyses for 30-Day Mortality

The results of the univariable Cox regression analyses for 30-day mortality are presented in Table 4. In the univariable analysis, pancreaticobiliary tumor type (*p* < 0.001), mGPS = 2 (*p* < 0.001), PNI < 40 (*p* = 0.008), CALLY < 67 (*p* < 0.001), CAR ≥ 12.2 (*p* < 0.001), and SII ≥ 1532 (*p* = 0.017) were associated with an increased risk of mortality. When metastatic distribution was examined, patients with multiple-organ involvement had a higher mortality risk than those with liver-only metastasis (*p* = 0.026). By contrast, sex, BMI, ECOG performance status, previous ERCP, and history of surgery showed no significant association with 30-day mortality (all *p* > 0.05).

**Figure 1:**
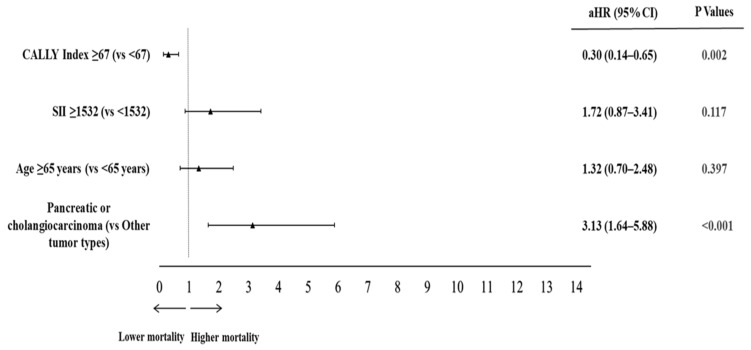
Multivariable Cox regression for 30-day mortality. Abbreviations: CALLY, CALLY index; SII, systemic immune-inflammation index. Footnote: Hazard ratios and 95% confidence intervals were derived from a clinically adjusted multivariable Cox proportional hazards model for 30-day mortality. Variables included in the final model are shown in the figure. In the figure, the pancreatic/cholangiocarcinoma category corresponds to pancreaticobiliary tumors, including pancreatic cancer, cholangiocarcinoma, and gallbladder cancer.

In the multivariable Cox analysis, pancreaticobiliary tumor type was independently associated with an increased risk of 30-day mortality (*p* < 0.001), whereas CALLY ≥ 67 was independently associated with a lower mortality risk (*p* = 0.002). In contrast, SII ≥ 1532 and age ≥ 65 were not independently associated with mortality (*p* = 0.117 and *p* = 0.397, respectively). The results are presented in Figure 1.

### 3.4. Risk Stratification Using CALLY Index and mGPS

Using the combined CALLY index and mGPS for risk stratification revealed marked differences in 30-day mortality across groups. The 30-day mortality rate was 8.0% in the low-risk group (CALLY ≥ 67 and mGPS 0–1) and 44.1% in the high-risk group (CALLY < 67 and mGPS 2). The intermediate-risk group included the remaining discordant combinations of CALLY and mGPS and had an overall 30-day mortality rate of 13.5%. Within this intermediate category, patients with CALLY ≥ 67 and mGPS 2 constituted the majority of cases (*n* = 42) and had a 30-day mortality rate of 14.3%, whereas patients with CALLY < 67 and mGPS 0–1 accounted for 10 cases and had a 30-day mortality rate of 10.0%. These findings are presented in Table 5.

## 4. Discussion

In this study, the association between preprocedural CALLY index and mGPS and early mortality was evaluated in patients with advanced malignant biliary obstruction undergoing palliative PTBD. Our findings demonstrated that the CALLY index was independently associated with 30-day mortality after PTBD, and that a risk stratification based on the combined use of CALLY and mGPS could meaningfully discriminate patients in terms of early mortality risk. In this context, the CALLY–mGPS model may provide a practical risk stratification approach that could contribute to clinical management planning in the early post-PTBD period.

Inflammation, immune function, and serum albumin are the three core components driving variation in the CALLY index, and they influence prognosis in patients with cancer through distinct yet interconnected biological pathways. The relationship between inflammation and tumor development is multifaceted. An inflammatory tumor microenvironment may support biological processes that enhance malignant cell proliferation, local invasion, and metastatic capacity. Pro-inflammatory cytokines and chemokines released by inflammatory cells not only promote tumor progression but may also impair immune effector functions—for example, by suppressing T-cell activation and expansion and thereby weakening antitumor immunity [20]. Host immune competence is likewise critical for cancer outcomes: T cells and natural killer (NK) cells exert antitumor effects by recognizing and eliminating malignant cells. However, tumor cells can evade immune surveillance through multiple mechanisms, including downregulation of major histocompatibility complex (MHC) expression and induction of immunosuppressive cell populations such as regulatory T cells and myeloid-derived suppressor cells [21]. Serum albumin, in turn, is an important marker that reflects not only nutritional status but also systemic inflammatory burden and hepatic reserve; low albumin levels are commonly associated with malnutrition, inflammation, and hepatic dysfunction, and hypoalbuminemia is linked to adverse outcomes as a surrogate of physiological stress related to tumor burden [18]. Collectively, these three dimensions—systemic inflammation, immune competence, and nutritional reserve—interact to shape the CALLY index and provide an integrated representation of key determinants of prognosis in patients with cancer.

Cut-off values reported for the CALLY index vary across studies, largely due to differences in calculation units and scaling, which limit direct comparability. In this context, Chen et al. reported in their meta-analysis of gastrointestinal malignancies that low CALLY levels were consistently associated with poor prognosis, despite differences in the cut-off values used across studies [18]. In line with this evidence, the CALLY index retained an independent association with 30-day mortality in our multivariable analysis. Patients with lower CALLY values had a higher risk of early mortality following PTBD. Based on our current review of the literature, no prior study has directly examined the association between the CALLY index and 30-day mortality in patients with malignant biliary obstruction treated with PTBD. In this respect, our study provides original data on the potential role of CALLY in estimating early mortality risk in this patient population. Although several indices, including SII, CAR, mGPS, and PNI, were associated with early mortality in univariable analyses, only CALLY retained its prognostic significance in the multivariable model. This finding may be related to the composite structure of the CALLY index, which captures inflammatory response, nutritional reserve, and immune status at the same time. In contrast, shared components and the resulting collinearity among the other indices may have limited their independent contribution in the multivariable model. Overall, the CALLY index may provide more consistent and clinically meaningful prognostic information for early risk stratification in this high-risk population undergoing PTBD. However, because metastatic burden showed collinearity with systemic inflammation- and nutrition-based indices, the independent prognostic role of CALLY should be interpreted with caution. CALLY may not be entirely independent of tumor burden; rather, it may partly reflect the host inflammatory and nutritional consequences of advanced metastatic disease. Therefore, future studies incorporating more detailed measures of metastatic burden are needed to clarify whether CALLY provides prognostic information beyond tumor extent alone.

In line with the integrative nature of CALLY described above, the CRP- and albumin-based mGPS has also been supported by strong evidence as a prognostic indicator in patients with cancer. In a meta-analysis evaluating the prognostic value of mGPS in pancreatic cancer, Zhang et al. demonstrated that higher mGPS was associated with poorer prognosis [22]. Consistently, in our study, increasing mGPS levels were associated with higher 30-day mortality, in agreement with the existing literature. However, obstructive jaundice and concomitant cholangitis may elevate CRP levels and reduce serum albumin concentrations independent of tumor biology. Therefore, biliary obstruction itself may have influenced the mGPS distribution in the present cohort, in which 68.2% of patients were classified as mGPS 2.

In our study, the 30-day mortality rate after PTBD was 25.1%. This rate was higher than the 19.8% 30-day mortality reported in the referenced multicenter observational study [9]. Reported 30-day mortality rates in the literature vary widely depending on center characteristics, patient selection, and clinical severity, ranging from approximately 10% in some series [23], to over 20% in cohorts with malignant biliary obstruction [24,25]. These differences may be related to factors such as performance status, comorbidity burden, tumor type and extent, and the presence of concomitant infection or clinical instability. In line with this, pancreaticobiliary tumor type was independently associated with an increased risk of early mortality in our cohort. Previous studies of biliary drainage in malignant biliary obstruction have similarly reported pancreatic cancer as one of the groups with the poorest short-term outcomes and higher early mortality [23]. Accordingly, the persistence of pancreaticobiliary tumor type as an independent risk determinant in our study aligns with the existing evidence. The observed differences in primary tumor distribution between survivors and non-survivors may also reflect the biological heterogeneity of malignant biliary obstruction, as tumor type can influence biliary obstruction patterns, systemic inflammatory burden, disease extent, and short-term mortality risk.

Although ECOG ≥2 was observed more often in patients who died within 30 days than in those who survived, this variable was not statistically significant in the univariable Cox regression analysis. This finding may be attributable, at least in part, to the small number of early mortality events and the limited 30-day follow-up window, both of which may have reduced statistical power. In addition, ECOG performance status may overlap with other indicators of advanced disease, systemic inflammation, nutritional impairment, tumor burden, and clinical frailty. Therefore, collinearity or shared prognostic information between ECOG and these variables may have attenuated its statistical significance in this cohort.

Consistent with effective biliary decompression after PTBD, our cohort showed a marked improvement in cholestatic parameters by day 7, with significant declines in both total and direct bilirubin (*p* < 0.001 for both). Similar early reductions in bilirubin within the first week after PTBD have also been reported in the literature; for example, Heedman et al. observed a decrease from 237 µmol/L to 180 µmol/L during the first week [26]. Concordant decreases in ALP/GGT and transaminases further supported relief of obstruction. In contrast, increases in CRP and leukocyte/neutrophil counts suggest a measurable post-procedural inflammatory response, which may reflect intervention-related inflammation, physiological stress, or early infectious complications. Septic complications were available in the clinical records; however, their distribution across CALLY–mGPS risk groups was not evaluated in a predefined formal analysis. Therefore, post-procedural inflammatory markers should be interpreted cautiously in the early period after PTBD. In line with this, post-procedural CALLY values were not evaluated because the primary aim of this study was preprocedural risk stratification, and post-PTBD CRP levels may be influenced by procedure-related inflammatory changes.

In a previous study of patients undergoing PTBD for malignant biliary obstruction, a risk classification based on PNI and NLR was reported to be associated with short-term survival [27]. In contrast, a risk stratification combining CALLY and mGPS has not been previously described in the literature. In our study, the CALLY–mGPS-based classification yielded 30-day mortality rates of 8.0% in the low-risk group, 13.5% in the intermediate-risk group, and 44.1% in the high-risk group, indicating a clear separation of patients with the poorest early outcomes. This marked absolute difference suggests that tailoring early post-PTBD surveillance intensity and supportive care strategies according to risk level may assist clinical decision-making and provides a practical approach for patient management.

The retrospective, single-center design and the heterogeneous cohort including multiple primary tumor types are limitations of this study. The inclusion of multiple primary tumor types may limit the generalizability of the CALLY–mGPS model, as different malignancies may have distinct biological behavior, inflammatory profiles, biliary obstruction patterns, and short-term mortality risks. In addition, the ROC-derived CALLY cut-off of 67 was data-driven and generated within the same cohort in which its prognostic performance was evaluated; therefore, optimism bias cannot be excluded, and this threshold may not be directly transferable to external cohorts without recalibration. ECOG performance status was obtained retrospectively from clinical records, which may have introduced information bias. Moreover, data on comorbidity burden, such as the Charlson Comorbidity Index, were not available; therefore, the potential effect of comorbid conditions on early procedural mortality could not be fully assessed. Nevertheless, our analysis provides real-world data from patients who underwent PTBD at a tertiary referral center. Future multicenter prospective studies are needed to validate these findings, perform tumor type-specific subgroup analyses, recalibrate the CALLY cut-off if necessary, and prospectively evaluate the CALLY/CALLY–mGPS approach.

## 5. Conclusions

This study suggests that preprocedural CALLY and mGPS values may offer clinically useful information for assessing the risk of early mortality in patients with advanced disease undergoing PTBD. In particular, the independent association between the CALLY index and 30-day mortality suggests a discriminative contribution to early post-procedural risk stratification. Both CALLY and mGPS are derived from routine laboratory parameters and are readily applicable in clinical practice. This study provides, to date, the first evidence on the prognostic value of the CALLY index in patients with malignant biliary obstruction treated with palliative PTBD and shows that a CALLY–mGPS-based classification can differentiate 30-day mortality risk. This approach may aid in tailoring post-PTBD monitoring intensity and supportive care strategies according to individual risk.

## Figures and Tables

**Table 1:** Baseline characteristics according to 30-day mortality after PTBD.

Characteristic	Total (*n* = 179)	No 30-Day Mortality (*n* = 134, 74.9%)	30-Day Mortality (*n* = 45, 25.1%)
**Demographic Characteristics**			
Female sex, No. (%)	83 (46.4)	60 (44.8)	23 (51.1)
Male sex, No. (%)	96 (53.6)	74 (55.2)	22 (48.9)
BMI ≤ 22.5 kg/m^2^, No. (%)	67 (37.4)	51 (38.1)	16 (35.6)
BMI > 22.5 kg/m^2^, No. (%)	112 (62.6)	83 (61.9)	29 (64.4)
ECOG 0–1, No. (%)	107 (59.8)	98 (73.1)	9 (20.0)
ECOG ≥ 2, No. (%)	72 (40.2)	36 (26.9)	36 (80.0)
**Primary Tumor Type, No. (%)**			
Pancreatic	65 (36.3)	55 (41.0)	10 (22.2)
Cholangiocarcinoma/gallbladder	47 (26.3)	41 (30.6)	6 (13.3)
Colorectal	13 (7.3)	10 (7.5)	3 (6.7)
Gastric	27 (15.1)	16 (11.9)	11 (24.4)
Other *	27 (15.1)	12 (9.0)	15 (33.3)
**Baseline Laboratory Findings**			
Albumin ≥ 30.0 g/L, No. (%)	95 (53.1)	80 (59.7)	15 (33.3)
Albumin < 30.0 g/L, No. (%)	84 (46.9)	54 (40.3)	30 (66.7)
Total bilirubin < 12.65 mg/dL, No. (%)	79 (44.1)	61 (45.5)	18 (40.0)
Total bilirubin ≥ 12.65 mg/dL, No. (%)	100 (55.9)	73 (54.5)	27 (60.0)
**Inflammatory and Nutritional Indices**			
mGPS 0–1, No. (%)	57 (31.8)	53 (39.6)	4 (8.9)
mGPS 2, No. (%)	122 (68.2)	81 (60.4)	41 (91.1)
PNI ≥ 40, No. (%)	68 (38.0)	60 (44.8)	8 (17.8)
PNI < 40, No. (%)	111 (62.0)	74 (55.2)	37 (82.2)
CALLY ≥ 67, No. (%)	92 (51.4)	82 (61.2)	10 (22.2)
CALLY < 67, No. (%)	87 (48.6)	52 (38.8)	35 (77.8)
CAR < 12.2, No. (%)	90 (50.3)	78 (58.2)	12 (26.7)
CAR ≥ 12.2, No. (%)	89 (49.7)	56 (41.8)	33 (73.3)
SII < 1532, No. (%)	89 (49.7)	74 (55.2)	15 (33.3)
SII ≥ 1532, No. (%)	90 (50.3)	60 (44.8)	30 (66.7)
**Procedural and Treatment-Related Characteristics**			
Prior ERCP performed, No. (%)	30 (16.8)	25 (18.7)	5 (11.1)
No prior ERCP, No. (%)	149 (83.2)	109 (81.3)	40 (88.9)
History of surgery, No. (%)	53 (29.6)	40 (29.9)	13 (28.9)
No history of surgery, No. (%)	126 (70.4)	94 (70.1)	32 (71.1)
Chemotherapy before PTBD, No. (%)	66 (36.9)	43 (32.1)	23 (51.1)
No chemotherapy before PTBD, No. (%)	113 (63.1)	91 (67.9)	22 (48.9)
Chemotherapy discontinued after PTBD, No. (%)	96 (53.6)	59 (44.0)	37 (82.2)
Chemotherapy continued after PTBD, No. (%)	83 (46.4)	75 (56.0)	8 (17.8)
**Chemotherapy regimen prior to PTBD**			
No systemic chemotherapy	43 (24.0)	26 (19.4)	16 (35.6)
Fluoropyrimidine-based doublet	58 (32.4)	43 (32.1)	15 (33.3)
Gemcitabine-based regimens	34 (19.0)	30 (22.4)	4 (8.9)
Intensified triplet regimens (FOLFIRINOX-based)	26 (14.5)	25 (18.7)	1 (2.2)
Other regimens	18 (10.1)	10 (7.5)	8 (17.8)
**Metastatic Disease Pattern**			
**Liver-only metastasis**	**102 (57.0)**	**72 (53.7)**	**30 (66.7)**
**Non-liver metastasis only**	**39 (21.8)**	**33 (24.6)**	**6 (13.3)**
**Liver + extrahepatic metastasis**	**28 (15.6)**	**24 (17.9)**	**4 (8.9)**
**Multiple metastatic sites (≥2 organs)**	**10 (5.6)**	**5 (3.7)**	**5 (11.1)**

* Other includes breast, ovarian, hepatocellular carcinoma, and cancers of unknown primary. Abbreviations: BMI, body mass index; CAR, C-reactive protein–to–albumin ratio; ECOG, Eastern Cooperative Oncology Group performance status; ERCP, endoscopic retrograde cholangiopancreatography; mGPS, modified Glasgow Prognostic Score; PNI, Prognostic Nutritional Index; PTBD, percutaneous transhepatic biliary drainage; SII, systemic immune-inflammation index. Footnote: Values are presented as number (percentage). Post-PTBD 30-day mortality was evaluated as death recorded during the first 30 days after the procedure. All variables were assessed using baseline clinical and laboratory data obtained prior to PTBD.

**Table 2:** Discriminative performance of inflammatory and nutritional indices for 30-day mortality.

Index	AUC (95% CI)	Optimal Cut-Off	Sensitivity %	Specificity %	PPV %	NPV %	Accuracy %	Youden Index
CAR	0.66 (0.57–0.74)	≥12.2	73.3	58.2	37.1	86.7	62.0	0.315
SII	0.64 (0.56–0.72)	≥1532	66.7	55.2	33.3	83.1	58.1	0.219
CALLY index	0.72 (0.64–0.80)	<67	77.8	61.2	40.2	89.1	65.4	0.390

Abbreviations: AUC, area under the receiver operating characteristic curve; CI, confidence interval; CAR, C-reactive protein–to–albumin ratio; SII, systemic immune-inflammation index; PPV, positive predictive value; NPV, negative predictive value.

**Table 3:** Laboratory parameters before and after PTBD (paired analysis).

Parameter	Pre-PTBD (Mean ± SD)	Post-PTBD (Mean ± SD)	*p* Value
WBC (×10^3^/µL)	9.41 ± 4.55	11.52 ± 6.38	<0.001
Neutrophils (/µL)	7777 ± 6672	9244 ± 5991	0.014
Lymphocytes (/µL)	1196 ± 658	1304 ± 949	0.063
Total bilirubin (mg/dL)	13.82 ± 7.03	7.36 ± 5.42	<0.001
Direct bilirubin (mg/dL)	10.42 ± 5.49	5.51 ± 3.99	<0.001
ALT (U/L)	111.3 ± 91.4	55.8 ± 47.0	<0.001
AST (U/L)	122.4 ± 100.7	70.0 ± 62.6	<0.001
GGT (U/L)	561.1 ± 407.9	284.5 ± 251.9	<0.001
ALP (U/L)	685.6 ± 527.1	367.2 ± 279.9	<0.001
CRP (mg/L)	59.6 ± 61.0	102.9 ± 83.3	<0.001

Abbreviations: ALP, alkaline phosphatase; ALT, alanine aminotransferase; AST, aspartate aminotransferase; CRP, C-reactive protein; GGT, gamma-glutamyl transferase; PTBD, percutaneous transhepatic biliary drainage; SD, standard deviation; WBC, white blood cell count. Footnote: Paired comparisons were performed using paired-samples *t*-test or Wilcoxon signed-rank test, as appropriate. Post-PTBD day-7 laboratory measurements were available only for patients who were alive and had repeat sampling at that time point; therefore, the paired pre/post-PTBD analysis does not include patients who died early after the procedure.

**Table 4:** Univariable Cox proportional hazards analysis for 30-day mortality.

Variable	Comparison (High-Risk vs. Reference)	HR (95% CI)	*p* Value
**Sex**	Female vs. male	0.78 (0.39–1.53)	0.461
**BMI**	≤22.5 vs. >22.5 kg/m^2^	1.11 (0.55–2.25)	0.768
**ECOG**	≥2 vs. 0–1	1.47 (0.79–2.74)	0.223
**Pancreaticobiliary tumors**	**vs. others**	**3.45 (1.85–6.67)**	**<0.001**
**mGPS**	**2 vs. 0–1**	**6.71 (2.27–19.82)**	**<0.001**
**PNI**	**<40 vs. ≥40**	**2.83 (1.40–5.72)**	**0.008**
**CALLY**	**<67 vs. ≥67**	**3.70 (1.95–7.01)**	**<0.001**
**CAR**	**≥12.2 vs. <12.2**	**3.83 (1.82–8.07)**	**<0.001**
**SII**	**≥1532 vs. <1532**	**2.47 (1.22–5.00)**	**0.017**
**Prior ERCP**	Yes vs. no	1.84 (0.66–5.12)	0.244
**History of surgery**	Yes vs. no	1.05 (0.50–2.20)	0.909
**Metastatic site**	Non-liver only vs. liver-only	0.54 (0.22–1.31)	0.176
	Liver + extrahepatic vs. liver-only	1.42 (0.67–3.01)	0.357
	**Multiple sites vs. liver-only**	**2.61 (1.12–6.06)**	**0.026**

Abbreviations: BMI, body mass index; CAR, C-reactive protein–to–albumin ratio; ECOG, Eastern Cooperative Oncology Group performance status; ERCP, endoscopic retrograde cholangiopancreatography; HR, hazard ratio; mGPS, modified Glasgow Prognostic Score; PNI, Prognostic Nutritional Index; SII, systemic immune-inflammation index. Footnote: Hazard ratios were calculated using separate univariable Cox proportional hazards models for each variable. Thirty-day mortality was evaluated as death recorded within the first 30 days after PTBD. All variables were derived from baseline clinical and laboratory data obtained prior to PTBD. Pancreaticobiliary tumors included pancreatic cancer, cholangiocarcinoma, and gallbladder cancer.

**Table 5:** Risk stratification based on the combined CALLY index and Glasgow Prognostic Score.

Risk Group	Definition	*n*	30-Day Mortality, *n* (%)
**Low**	CALLY ≥ 67 & mGPS 0–1	50	4 (8.0)
**Intermediate**	**Other combinations**	**52**	**7 (13.5)**
**-Subgroup A**	CALLY ≥ 67 & mGPS 2	42	6 (14.3)
**-Subgroup B**	CALLY < 67 & mGPS 0–1	10	1 (10.0)
**High**	CALLY < 67 & mGPS 2	77	34 (44.1)

Abbreviations: CALLY, CALLY index; mGPS, modified Glasgow Prognostic Score. Footnote: CALLY was dichotomized using a ROC-derived cut-off of 67 (<67 vs. ≥67); mGPS was categorized as 0–1 vs. 2.

## Data Availability

The datasets generated and/or analyzed during the current study are not publicly available due to institutional and patient privacy regulations but are available from the corresponding author upon reasonable request.

## References

[1] Sutter C.M., Ryu R.K. (2015). Percutaneous Management of Malignant Biliary Obstruction. Tech. Vasc. Interv. Radiol..

[2] Pu L.Z., Singh R., Loong C.K., de Moura E.G. (2016). Malignant Biliary Obstruction: Evidence for Best Practice. Gastroenterol. Res. Pract..

[3] Das M., van der Leij C., Katoh M., Benten D., Hendriks B.M.F., Hatzidakis A. (2021). CIRSE Standards of Practice on Percutaneous Transhepatic Cholangiography, Biliary Drainage and Stenting. Cardiovasc. Interv. Radiol..

[4] van Delden O.M., Laméris J.S. (2008). Percutaneous drainage and stenting for palliation of malignant bile duct obstruction. Eur. Radiol..

[5] Nagino M., Hayakawa N., Nimura Y., Dohke M., Kitagawa S. (1992). Percutaneous transhepatic biliary drainage in patients with malignant biliary obstruction of the hepatic confluence. Hepatogastroenterology.

[6] Leng J.-J., Zhang N., Dong J.-H. (2014). Percutaneous transhepatic and endoscopic biliary drainage for malignant biliary tract obstruction: A meta-analysis. World J. Surg. Oncol..

[7] Bang J.Y., Hawes R., Varadarajulu S. (2022). Endoscopic biliary drainage for malignant distal biliary obstruction: Which is better–endoscopic retrograde cholangiopancreatography or endoscopic ultrasound?. Dig. Endosc..

[8] Zhang G.Y., Li W.T., Peng W.J., Li G.D., He X.H., Xu L.C. (2014). Clinical outcomes and prediction of survival following percutaneous biliary drainage for malignant obstructive jaundice. Oncol. Lett..

[9] Turan A.S., Jenniskens S., Martens J.M., Rutten M., Yo L.S.F., van Strijen M.J.L., Drenth J.P.H., Siersema P.D., van Geenen E.J.M. (2022). Complications of percutaneous transhepatic cholangiography and biliary drainage, a multicenter observational study. Abdom. Radiol..

[10] Vandenabeele L.A.M., Dhondt E., Geboes K.P., Defreyne L. (2017). Percutaneous stenting in malignant biliary obstruction caused by metastatic disease: Clinical outcome and prediction of survival according to tumor type and further therapeutic options. Acta Gastroenterol. Belg..

[11] Zhou Y., Liu Z., Cheng Y., Li J., Fu W. (2024). Prognostic value of the modified Glasgow Prognostic Score in biliary tract cancer patients: A systematic review and meta-analysis. J. Gastrointest. Surg..

[12] Wu D., Wang X., Shi G., Sun H., Ge G. (2021). Prognostic and clinical significance of modified Glasgow Prognostic Score in pancreatic cancer: A meta-analysis of 4629 patients. Aging.

[13] He L., Li H., Cai J., Chen L., Yao J., Zhang Y., Xu W., Geng L., Yang M., Chen P. (2018). Prognostic value of the Glasgow prognostic score or modified Glasgow Prognostic Score for patients with colorectal cancer receiving various treatments: A systematic review and meta-analysis. Cell. Physiol. Biochem..

[14] Utsumi M., Inagaki M., Kitada K., Tokunaga N., Kondo M., Yunoki K., Sakurai Y., Hamano R., Miyasou H., Tsunemitsu Y. (2023). Preoperative C-reactive protein-to-albumin ratio as a prognostic factor in biliary tract cancer: A systematic review and meta-analysis. Medicine.

[15] Geng Y., Qi Q., Sun M., Chen H., Wang P., Chen Z. (2015). Prognostic nutritional index predicts survival and correlates with systemic inflammatory response in advanced pancreatic cancer. Eur. J. Surg. Oncol..

[16] Migita K., Takayama T., Saeki K., Matsumoto S., Wakatsuki K., Enomoto K., Tanaka T., Ito M., Kurumatani N., Nakajima Y. (2013). The prognostic nutritional index predicts long-term outcomes of gastric cancer patients independent of tumor stage. Ann. Surg. Oncol..

[17] Dong M., Shi Y., Yang J., Zhou Q., Lian Y., Wang D., Ma T., Zhang Y., Mi Y., Gu X. (2020). Prognostic and clinicopathological significance of systemic immune-inflammation index in colorectal cancer: A meta-analysis. Ther. Adv. Med. Oncol..

[18] Chen D., Ma Y., Li J., Wen L., Liu L., Su J., Wu J., Wang P., Zhang G., Huang C. (2025). Prognostic and clinicopathological significance of C-reactive protein–albumin–lymphocyte(CALLY) in patients with digestive system neoplasms: A systematic review and meta-analysis. World J. Surg. Oncol..

[19] Collins G.S., Reitsma J.B., Altman D.G., Moons K.G. (2015). Transparent reporting of a multivariable prediction model for individual prognosis or diagnosis (TRIPOD): The TRIPOD statement. BMJ.

[20] Elinav E., Nowarski R., Thaiss C.A., Hu B., Jin C., Flavell R.A. (2013). Inflammation-induced cancer: Crosstalk between tumours, immune cells and microorganisms. Nat. Rev. Cancer.

[21] Leone R.D., Powell J.D. (2020). Metabolism of immune cells in cancer. Nat. Rev. Cancer.

[22] Zhang H., Ren D., Jin X., Wu H. (2020). The prognostic value of modified Glasgow Prognostic Score in pancreatic cancer: A meta-analysis. Cancer Cell Int..

[23] Chan K., Vigneswaran G., Modi S., Sew Hee C., Maclean D., Stedman B., Bryant T., Maher B. (2025). Identifying predictive markers for survival in malignant biliary obstruction following percutaneous transhepatic biliary drainage. Clin. Radiol..

[24] Rees J., Mytton J., Evison F., Mangat K.S., Patel P., Trudgill N. (2020). The outcomes of biliary drainage by percutaneous transhepatic cholangiography for the palliation of malignant biliary obstruction in England between 2001 and 2014: A retrospective cohort study. BMJ Open.

[25] Sahinli H., Özet A. (2020). Prognostic and predictive factors in cancer patients with obstructive jaundice treated by percutaneous transhepatic biliary drainage: A single-center experience. J. Cancer Res. Ther..

[26] Heedman P.A., Åstradsson E., Blomquist K., Sjödahl R. (2018). Palliation of Malignant Biliary Obstruction: Adverse Events are Common after Percutaneous Transhepatic Biliary Drainage. Scand. J. Surg..

[27] Zakosek M., Bulatovic D., Pavlovic V., Filipovic A., Igic A., Galun D., Jovanovic D., Sisevic J., Masulovic D. (2022). Prognostic Nutritional Index (PNI) and Neutrophil to Lymphocyte Ratio (NLR) as Predictors of Short-Term Survival in Patients with Advanced Malignant Biliary Obstruction Treated with Percutaneous Transhepatic Biliary Drainage. J. Clin. Med..

